# Prevalence and Determinants of the Use of Antibiotics by Self-Medication in the Pediatric Population in Oujda, Morocco

**DOI:** 10.7759/cureus.60126

**Published:** 2024-05-12

**Authors:** Hasnae Elhaddadi, Amal Hamami, Anane Sara, Aziza Elouali, Abdeladim Babakhouya, Maria Rkain

**Affiliations:** 1 Department of Pediatrics, Mohammed VI University Hospital, Oujda, MAR; 2 Faculty of Medicine and Pharmacy, Mohammed 1st University, Oujda, MAR

**Keywords:** antimicrobial resistance, morocco, self-medication, child, antibiotics

## Abstract

Objective: Antibiotic resistance driven by antibiotic self-medication and inappropriate use of antibiotics is a growing global health threat. Our study aimed to describe parents' self-medication practices with antibiotics, determine the factors favoring their use in the pediatric population, and assess parents' knowledge of the role of antibiotics and antimicrobial resistance.

Materials and methods: We conducted a cross-sectional study over two months (September and October 2023). Data collection was performed using a questionnaire-guided interview. We included 460 parents of children consulting or hospitalized in the Pediatric Department of the University Hospital Mohammed VI in Oujda, Morocco.

Results: A total of 62% of parents questioned were mothers. Self-medication with antibiotics was noted in 313 families (68%). Parents used antibiotics mainly to treat cough (43%) and fever (24%). Betalactams were the antibiotic class most used by parents (72%). Information on dosage and methods of antibiotic administration was provided by pharmacy staff but was not respected by most parents (78%). Efficacy of treatment (58%), saving consultation costs and time (47%), and the ease of obtaining treatment without a prescription (42%) were reported in the majority of cases as reasons for using antibiotics by self-medication. In our context, the probability of self-medicating with antibiotics in the pediatric population is increased by female gender (OR=1.04), low level of education (OR=1.02), low socio-economic status (OR=1.09), and buying antibiotics without a prescription (OR=1.22).

Conclusion: Although antibiotic self-medication in children is a worldwide phenomenon, influenced by several geographical, cultural, and economic factors, there is an urgent need to promote a global health strategy.

## Introduction

Self-medication is a phenomenon whose definition is much more complex than the one currently proposed [1]. According to the World Health Organization (WHO), self-medication is the use of a medicine by an individual, on his or her initiative or that of a person close to him or her, to treat a self-identified affection or symptom, without recourse to a health professional [2]. The WHO has pointed out that in order to achieve rational use of medicines, it is necessary to use appropriate medications that can treat patients' clinical symptoms in specific geographical areas with fewer complications and costs [3].

The discovery of antibiotics revolutionized medicine in the 20th century, with major discoveries such as sulfonamide in 1935 and penicillin after the Second World War. However, bacterial resistance quickly tempered the initial hope of definitive control of infectious diseases [4]. Unlike other drugs, self-medication with antibiotics is characterized by unjustified indications, inappropriate choice of antibiotic, use of insufficient doses, and inadequate duration of treatment. These practices can lead to the inappropriate use of these drugs by self-medication, leading to the emergence of bacterial resistance [2].

Antimicrobial resistance (AMR) is currently a major and growing health problem, ranked by the WHO as one of the world's 10 major public health challenges. Overuse of antibiotics increases the selection pressure of mutated strains of microbes, accelerating the development of AMR [5]. In the first global report on antibiotic resistance presented in 2014 in Geneva and covering 114 countries, the WHO warned the following: "Unless we take significant steps to improve infection prevention efforts and also change the way we produce, prescribe, and use antibiotics, the world will lose more and more of these global public health assets, and the implications will be devastating" [6].

Our study aimed to describe parents' self-medication practices with antibiotics, determine the factors favoring their use in the pediatric population, and assess parents' knowledge of the role of antibiotics and antimicrobial resistance.

## Materials and methods

Study design

A cross-sectional study based on a direct interview with parents spread over two months during 2023 (September and October). The study was conducted at the Mother-Child Hospital of the Mohammed VI University Hospital in Oujda and Mohammed I University in Oujda, Morocco. The study combined a descriptive method based on direct interviews with parents and an analytical method using statistical software.

Study population

The pediatrics department, pediatric emergencies, and specialized pediatric consultation units of the Mohammed VI University Hospital in Oujda receive a varied population from all over the eastern region of Morocco. Parents of children hospitalized in the pediatric department, consulting the pediatric emergency department, or the specialized pediatric consultation units were invited to participate in the interview, regardless of the reason for their consultation. Random sampling was used. The study population consisted of 460 parents. The inclusion criteria were to be a parent (mother or father) and to have a child under 16 years of age who agreed to participate in our study after verbal consent.

Questionnaire

The interview with the parents was carried out in optimal conditions of listening, confidentiality, and comfort. The first part of the data enabled us to collect the socio-demographic characteristics of the parents (age, level of education, socio-economic level, number of children, type of health coverage, etc.). In the second part of the data, we sought to assess knowledge and attitudes towards the use of antibiotics (definition of antibiotics and their role, knowledge of side effects/dangers linked to antibiotic abuse, multi-resistant infections, and antimicrobial resistance). The third part of the data focused on self-medication with antibiotics in the last 12 months and the factors that encourage this self-medication. Participation was voluntary. The anonymity and confidentiality of their answers were assured.

Data analysis

Data were encoded with SPSS version 22 (Armonk, NY: IBM Corp.). Categorical variables were expressed as frequencies, while numerical variables (age, etc.) were presented as means. The existence of associations and comparisons between qualitative variables were highlighted Chi-squared tests. Comparisons between quantitative variables were made using analysis of variance. To identify the factors involved in antibiotic self-medication, logistic regressions were performed. Univariate logistic regression models were applied to obtain the odds ratio (OR). For multiple logistic regression, variables with a p-value > 0.05 were excluded. The corresponding p-value for the various statistical tests was considered significant for p < 0.001.

## Results

Socio-demographic characteristics

Out of a total of 460 families included in this study, mothers were respondents in the majority of cases (n=285, 62%), most of whom were housewives (n=236, 83%). A small percentage of parents were uneducated (n=105, 23%), and only (n=50) 11% had a university degree. The majority of families had a low socio-economic status (n=299, 65%). Families were divided between urban (n=331, 72%) and rural (28%) living environments. Most parents interviewed (n=322, 70%) had two or more children (Table 1).

**Table 1:** Socio-demographic characteristics of participating parents in our study (n=460).

Variables		Number (n)	Percentage %
The parent	Father	175	38%
	Mother	285	62%
Socio-economic level	Favorable	161	35%
	Unfavorable	299	65%
Educational level	Illiterate	106	23%
	Primary level	46	10%
	Secondary level	258	56%
	University level	50	11%
Health coverage	Yes	345	75%
	No	115	25%
Mother's occupation	Housewife	382	83%
	Civil servant	78	17%
Living environment	Rural	129	28%
	Urban	331	72%
Number of children	1 child	138	30%
	2 or more children	322	70%

Symptoms causing self-medication with antibiotics

**Figure 1:**
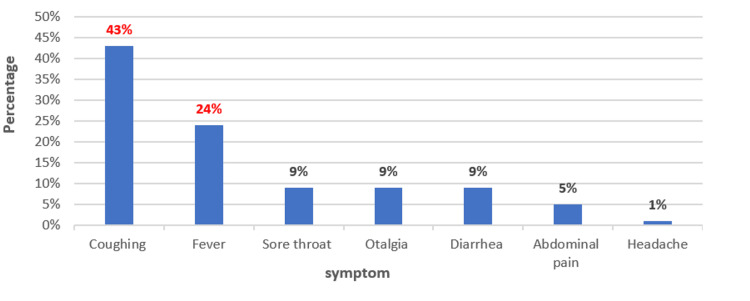
Symptoms treated with antibiotics by parents who reported using antibiotics without a doctor's prescription in our study (n=313).

Self-medication with antibiotics was noted in 313 families (68%) and encouraged by mothers (n=289, 63%) more than fathers. Self-medication with antibiotics was more frequent in two to five years (n=184, 40%) and >5-15 years (n=115, 25%) age groups. According to the majority of parents surveyed (n=358, 78%), all respiratory infections are treated with antibiotics. Self-medication with antibiotics in children was used to treat several symptoms, including cough (n=198, 43%), fever (n=110, 24%), sore throat (n=41, 9%), earache (n=41, 9%), diarrhea (n=41, 9%), abdominal pain (n=24, 5%), and headache (n=5, 1%) (Figure 1). Betalactams were the antibiotic class most used by parents (n=331, 72%), followed by trimethoprim-sulfamethoxazole (n=69, 15%) (Table 2). Information on dosage, duration, and methods of antibiotic administration was provided by pharmacy staff in the majority of self-medication antibiotic purchases (n=312, 68%) but was not respected by most parents (n=243, 78%). According to parents, the main reasons for non-compliance were that the child's general health had improved and that they had forgotten to take the treatment.

**Table 2:** Types of antibiotics self-medicated by parents in our study (n=313).

Type of antibiotic	Number (n)	Percentage %
Amoxicillin-clavulanic acid	150	48%
Amoxicillin	75	24%
Trimethoprim-sulfamethoxazole	46	15%
Azithromycin	26	08%
Unspecified	16	05%
Total	313	100%

Parents' knowledge and perceptions of antibiotics and multi-resistant infections

In 85% of cases, parents were aware of the notion of the power of antibiotics as an anti-infectious treatment, but 69% of them gave a false definition of the role of antibiotics. The majority of parents in our study (n=294, 64%) had heard of the term antibiotic resistance or multidrug-resistant infections, and half of them said they would minimize the use of antibiotics by self-medication, or only take them when necessary and when prescribed by a doctor (Table 3).

**Table 3:** Parents' knowledge and perceptions of antibiotics and antibiotic resistance (n=460).

Parents' knowledge of antibiotics	Percentage %	
	Correct answer	Incorrect answer
Exact definition of the role of antibiotics	72%	28%
Antibiotics are very powerful medicines	82%	18%
Antibiotic duration must be respected	67%	33%
Health risks of stopping antibiotics during treatment	65%	35%
Antibiotics can have side effects	42%	58%
Exact definition of bacterial resistance to antibiotics	72%	28%
The existence of health risks associated with taking antibiotics self-medication	52%	48%

In Table 4, the educational level variable has been changed to a binary variable. Low educational level includes those with no education at all, those with less than primary education, and those who have completed primary education. The high educational level includes those with secondary and university education. There are statistically significant associations between the high educational level of parents practicing antibiotic self-medication and female gender, health insurance coverage, and knowledge of the role of antibiotics and bacterial resistance. On the other hand, low educational level was significantly associated with the use of antibiotics available at home.

**Table 4:** Statistically significant associations between low educational level and other variables among parents self-medicating with antibiotics (n=460). *Degrees of freedom=1. P-value <0.05 is considered statistically significant.

Variables		Educational level		p-Value	Chi-squared test value*
		Low level (n=152)	High level (n=308)		
Gender	Male	50 (28.5%)	125 (71.4%)	<0.021	1.2331
	Female	102 (35.7%)	183 (64.2%)	<0.001	4.4536
Adherence to health coverage		82 (53.9%)	302 (98%)	<0.001	1.2254
Antibiotic use available at home		38 (25%)	12 (3.8%)	<0.001	3.3455
Exact definition of the role of antibiotics		28 (18.4%)	272 (88.3%)	<0.001	1.3498
Exact definition of bacterial resistance to antibiotics		24 (15.5%)	281 (91.2%)	<0.001	2.9008
Health risks of stopping antibiotics during treatment		92 (59.7%)	301 (97%)	<0.001	3.3464
Health risks of self-medicating with antibiotics		124 (81.5%)	283 (91.8%)	<0.001	2.6574

Factors favoring self-medication with antibiotics

The reasons for self-medication cited by the group of parents who used antibiotics without medical advice are presented in Table 5. Efficacy of treatment, saving consultation costs, saving time, and the ease of obtaining treatment without a prescription were reported in the majority of cases. Therefore, it was noted that parents had very little awareness of engaging in self-medication when reusing old prescriptions prescribed by doctors or antibiotics obtained on the advice of pharmacy staff.

**Table 5:** Factors favoring self-medication with antibiotics among parents participating in our study (n=460).

Factors	Number (n)	Percentage %
Treatment efficacy	181	58%
Saving on consultation costs and time	114	47%
Easy to obtain treatment without a prescription	131	42%
Treatment recommended by pharmacy staff	93	30%
Availability of treatment at home	38	12%
Availability of treatment at home	25	08%
Similarity of symptoms with a previous infection-treated antibiotic treatment	15	05%

The factors significantly associated with antibiotic self-medication are shown in Table 6. The reference groups were made up of parents who had self-medicated with antibiotics in the 12 months before our study, and parents who said they had never self-medicated with antibiotics. The probability of self-medicating with antibiotics in the pediatric population in our context is increased by female gender, low level of education, low socio-economic status, and buying antibiotics without a prescription. On the other hand, the following factors reduce the likelihood of self-medicating with antibiotics having health coverage, defining bacterial resistance to antibiotics accurately, and perceiving that there are health risks when self-medicating with antibiotics.

**Table 6:** Factors associated with the likelihood of antibiotic self-medication in our study. *Significant positive association between the variable and antibiotic self-medication in the pediatric population.

Variables	Odds ratio (OR)	95% CI
Advanced age (year)	1.04^*^	1.02-1.06%
Gender (female versus male)	1.04^*^	1-1.08%
Health coverage (yes versus no)	0.58	0.45-0.73%
Educational level	1.02^*^	0.89-1.52%
Low socio-economic status	1.09	0.94-1.24%
Ease of buying antibiotics without a prescription	1.22^*^	0.92-2.35%
Exact definition of the role of antibiotics	0.82	0.17-1.47%
The notion that antibiotics are powerful (yes versus no)	1.16	0.87-1.45%
Exact definition of bacterial resistance to antibiotics	0.84	0.69-0.99%
Existence of health risks associated with stopping current antibiotics (yes versus no)	0.90	0.65-1.15%
Stopping antibiotics when symptoms disappear (yes versus no)	0.78	0.58-0.98%
Health risks of self-medicating with antibiotics (yes versus no)	0.88	0.62-1.12%

## Discussion

In our study, more than half the parents (68%) reported having self-medicated their children with antibiotics in the previous year, indicating that self-medication is common in our context. Our results highlight the problem of antibiotic safety in Morocco. The risk of antibiotic self-medication reported in our study is similar to those observed in other countries [7]. The prevalence of parental self-medication was 53.1% in Brazil [8], 32.8% in Spain [9], and 24% in China [10], suggesting that parental self-medication is a global problem in both developed and developing countries.

The child's age may influence parental behavior, and encourage self-medication. Our study showed that in most cases, children who self-medicated with antibiotics were over two years of age, with results similar to those reported in the literature [11,12]. The systematic review by Bert et al. concluded that there was a significant association between the child's advanced age and antibiotic self-medication [13]. In our study, there was a significant increase in the risk of antibiotic self-medication if there were several siblings in the family. The study by Ortiz et al. concluded that parental experience with previous children could generate the necessary confidence and courage in parents to choose the medication themselves and treat their children by self-medication [9].

In the present study, 76% of cases reported self-medicating with antibiotics for symptoms that most frequently included cough, fever, and sore throat among our participants. Our results are similar to those of several previous studies in the literature [14-16]. The Jordanian study by Al-Tarawneh et al. showed that all cases of antibiotic use by self-medication were to treat viral infections not requiring antibiotic treatment [17]. Pharmacy sales staff and pharmacists were the main sources of information on the type of antibiotic, dose, duration, and method of use. Abdel-Qader et al. pointed out in their study that a large proportion of the population using antibiotics by self-medication relied on the advice of pharmacists [18].

Our results show that broad-spectrum penicillins accounted for most of the antibiotics consumed by self-medicated children, followed by trimethoprim-sulfamethoxazole and macrolides. Worldwide, similar results have been reported by Franchi et al. in Italy [19] and by Maltezou et al. in Greece [20]. The duration and modalities of antibiotic treatment were not respected by 78% of our participants. The study by Al-Tarawneh et al. showed that antibiotic treatment was used for three days or less in 30% of cases; however, 34% of parents tended to continue using medication until symptoms disappeared [17]. Indeed, several studies have found that exposure of bacteria to sub-therapeutic levels of antibiotics, and the use of antibiotics for insufficient or less than the recommended period, can lead to an increased risk of bacterial antimicrobial resistance [21,22].

Parents' lack of knowledge about the role and use of antibiotics influences self-medication behavior. In the present study, according to the majority of parents surveyed (78%), all respiratory infections and infections of the otorhinolaryngeal sphere are treated with antibiotics, but viral infections do not require antibiotic treatment [23]. Indeed, rational use of antibiotics can be achieved by using the right antibiotics at the right doses for the right duration [24]. Over 70% of parents have well-defined antibiotic resistance; however, they use antibiotics without a doctor's prescription. Furthermore, the lack of awareness of antimicrobial resistance was observed in the questionnaire data. In summary, there is a lack of information about antibiotic efficacy and bacterial resistance.

The main reasons for self-medication reported by parents in our study were treatment efficacy, saving consultation costs, time, and the ease of obtaining antibiotic treatment without a medical prescription. The Tunisian study by Mabrouk et al., which included 354 parents, concluded that the main reason for antibiotic self-medication in the pediatric population in 58.9% of cases was the fact that the same antibiotic was already prescribed to treat the same symptoms. In 57.5% of cases, the antibiotic used came from a previous prescription for the same child and was recommended by the pharmacist in 39.7% of cases [25].

In our study, the risk factors for self-medication were female gender, advanced age, low level of education, low socio-economic status, and buying antibiotics without a prescription. A tendency for females to self-medicate with antibiotics has been confirmed in several previous studies [26,17]. On the other hand, other articles have found no significant difference between males and females in self-medication behavior [27]. A low level of education and illiterate status have been identified as being significantly associated with a lack of knowledge about antibiotics and antibiotic resistance, and with a high rate of antibiotic self-medication in several studies, such as Eng et al.'s study [28]. The analytical study by You et al. showed that a higher level of education and family income were associated with adequate knowledge and rational use of antibiotics by parents [29].

The results of this study must be considered in the context of its limitations. Firstly, although this is a regional survey, our results may not reflect the risk of self-medication with antibiotics in the general population, as only a small sample of parents from the Oriental region participated in our study. Secondly, the possibility of selection bias cannot be ruled out, given that we selected our sample by chance. Finally, we included only a few predictors of self-medication in the survey. Parental self-medication with antibiotics may be influenced by other factors, such as income level and difficult access to healthcare centers.

## Conclusions

The socio-economic factors listed in our study, combined with the misconceptions parents may have about antibiotics and the ease with which these drugs can be obtained from pharmacies without a prescription, create an environment conducive to the inappropriate use of antibiotics, which can encourage the emergence and rapid spread of bacterial resistance. Interventions based on WHO recommendations may be feasible in our country's context and have a positive impact on reducing the prevalence of self-medication with antibiotics. In our study, we demonstrated that defining bacterial resistance to antibiotics accurately and recognizing that there are health risks associated with self-medication reduced the risk of self-medication with antibiotics. This justifies the need for educational campaigns aimed at the general public.
